# Age-Related Differences in Anxiety and Depression Diagnosis among Adults in Puerto Rico during the COVID-19 Pandemic

**DOI:** 10.3390/ijerph20115922

**Published:** 2023-05-23

**Authors:** Stephanie Cameron-Maldonado, Cynthia M. Pérez, Emma Fernández-Repollet, Andrea López-Cepero

**Affiliations:** 1 Department of Biostatistics and Epidemiology, Graduate School of Public Health, University of Puerto Rico-Medical Sciences Campus, San Juan 00936, Puerto Rico; 2 Center for Collaborative Research in Health Disparities, San Juan 00936, Puerto Rico; 3 Department of Epidemiology, Rollins School of Public Health, Emory University, Atlanta, GA 30322, USA

**Keywords:** mental health, depression, anxiety, COVID-19, young adults

## Abstract

Residents of Puerto Rico bear a significant burden of mental health disorders, which the COVID-19 pandemic may have exacerbated. However, age-specific data on these disorders during the pandemic in Puerto Rico are scarce. This study evaluated age-related differences in the self-reported diagnosis of depression and anxiety among adults ≥18 years residing in Puerto Rico during the pandemic. An anonymous online survey was administered from December 2020 to February 2021 via Google Forms to measure self-reported sociodemographic and behavioral characteristics and physician-diagnosed mental health disorders. Multivariable logistic regression models were conducted for each self-reported mental health diagnosis after adjusting for sex, education, income, marital status, chronic diseases, and smoking. Out of 1945 adults, 50% were aged 40 years and over. Nearly 24% of responders self-reported an anxiety diagnosis, whereas 15.9% reported depression. Compared to individuals 50 years and over, those 18–29 y, 30–39 y, and 40–49 y had significantly higher odds of an anxiety diagnosis (OR = 1.84, 95% CI = 1.34–2.55; OR = 1.50, 95% CI = 1.09–2.07; and OR = 1.37, 95% CI = 1.01–1.87, respectively). However, no association between age and depression diagnosis was found. Despite anxiety and depression being frequent disorders during the pandemic in this sample, younger adults bear a higher burden of anxiety. Further research is needed to allocate appropriate mental health resources during emergencies according to population subgroups.

## 1. Introduction

Two years after COVID-19 was declared a pandemic, its aftermath has created a parallel mental health crisis on a global scale [1]. The United States (US) Census Bureau reported that 42% of Americans aged 18 y or older were experiencing symptoms of anxiety and depression during the pandemic [2]. Other studies worldwide have also documented a high prevalence of anxiety and depression during the pandemic [3,4,5,6,7]. For instance, in China, it was found that one-third of adults in the general population reported anxiety symptoms, whereas 17% reported depressive symptoms [3]. In Brazil, the prevalence of anxiety and depression in adults was 19% and 22%, respectively [7]. This burden of mental health disorders worldwide is alarming given the documented negative impact of anxiety and depression on health and well-being [8,9,10,11]. These include lower job satisfaction and higher emotional exhaustion, poorer quality of life, and the risk of cardiovascular diseases [8,9,10,11]. Thus, monitoring the population’s mental health and identifying high-risk groups for early intervention and management is imperative. 

Residents of Puerto Rico, a US territory primarily comprised of Latinx persons (99.8% identifying as Latinx), already experienced a high burden of mental health disorders prior to the COVID-19 pandemic [12,13,14,15]. For example, a pre-pandemic (2014–2016) study conducted on a representative sample of adults on the island (18–64 y) documented that the most prevalent mental health disorders were depression and anxiety (12.5% and 10.4%, respectively) [14]. The population of Puerto Rico has endured environmental and sociopolitical stressors during the past few decades, which may negatively affect the population’s mental health [14]. A study evaluating the impact of Hurricane María on mental health among middle-aged adults in 2017 reported that 32% had elevated depressive symptoms and 64% elevated anxiety symptoms [15]. Subsequently, Puerto Rico residents were hit by a 6.4-magnitude earthquake in January 2020, followed by seismic activity that lasted the entire year. At the time of writing, since COVID-19 was declared a pandemic by the World Health Organization, a total of 1,139,243 confirmed COVID-19 cases and 5891 total deaths have been reported in Puerto Rico [16,17]. In early March 2020, the government of Puerto Rico implemented rigorous control measures to safeguard the health of its population, which included one of the strictest lockdowns, curfews, and quarantine mandates [18,19]. Thus, experiencing an additional stressor such as the COVID-19 pandemic within a short timeframe makes it imperative to ascertain the mental health status of adults in Puerto Rico and inform evidence-based practices to improve mental health outcomes. 

Studies outside of Puerto Rico have reported that, during the pandemic, younger adults were disproportionally affected by stress, depression, anxiety, and loneliness compared to the older adult population [20]. For instance, data from the Understanding American Study (UAS) suggest that younger adults have higher anxiety and depression symptoms than older adults [5]. However, the prevalence of depression and anxiety disorders among different age groups during the pandemic in Puerto Rico remains unknown. The first island-wide epidemiologic study in Puerto Rico on the prevalence of mental health disorders was conducted in 1987 and reported that 18.5% and 10.3% of young adults experienced depressive and anxiety disorders, respectively [21]. Another study conducted among a representative sample of adults on the island (18–64 y) prior to Hurricane María (2017), documented that 10% had major depressive disorder and 13% had an anxiety disorder, but no comparison across age groups was conducted [22]. Thus, given the scarcity of data on mental health disorders during the pandemic in Puerto Rico and the disproportionate burden that specific population subgroups (i.e., younger adults [23]) may encounter, we examined the associations between age and self-reported depression and anxiety diagnoses among adults residing in Puerto Rico during the pandemic. Such information is needed to identify high-risk populations and provide adequate resources in future public health emergencies.

## 2. Materials and Methods

### 2.1. Study Design and Participants 

In this secondary analysis, we used data from a previous cross-sectional study [24]. Briefly, in the original parent study, we collected data through an anonymous online survey that was disseminated through organizations; institutional newsletters; social network pages (i.e., Facebook, Twitter, and Instagram); and academic groups. No stratification or island-wide sampling procedures were conducted given the limited resources and restrictions during the COVID-19 pandemic. The Google Forms platform was used to collect data of interest. The online survey was available for the public from December 15, 2020 through February 15, 2021. 

The online survey was developed from a literature review. The scales selected were translated into Spanish (the dominant language used by residents of Puerto Rico) and revised by experts. For comprehensive purposes, the survey was piloted on a group of men and women (n = 7; men = 1; women = 6) of various age groups from 20 to 58 y. 

The first page of the survey included an information sheet providing details about the study. People who were interested in participating proceeded to answer questions regarding their age and place of residency to determine their eligibility. If someone was found to be ineligible, the platform informed them and terminated the process. Eligible individuals then continued to complete the study survey. The inclusion criteria were being 18 years or older, presently residing in Puerto Rico, having access to an electronic device to complete the web-based survey, and being able to answer the survey in Spanish. Thus, individuals younger than 18 years, residing outside of Puerto Rico, without access to an electronic device, and unable to answer the survey questions in Spanish were ineligible. Eligible participants who completed the online survey were encouraged to share the study’s information with their contacts. No compensation was given to participants for completing the survey. As previously reported, a total of 2233 surveys were submitted; of which, 21 responders declined to participate, and 32 were ineligible. A total of 235 surveys were further eliminated due to duplicates (reporting the same set of responses for all items). Thus, a total of 1945 responders completed the online survey and were included in the present analysis. Overall, the study had a response rate of 87% for those who successfully completed the online survey. The study was approved by the Institutional Review Board of the University of Puerto Rico, Medical Sciences Campus. 

### 2.2. Study Measures 

The online survey measured self-reported sociodemographic and behavioral characteristics and mental health disorders of depression and anxiety. 

#### 2.2.1. Age 

Survey respondents self-reported their age into one of the following categories: 18–29 y, 30–39 y, 40–49 y, 50–69 y, 70–79 y, and 80 y or older. The age groups were further collapsed due to small cell sizes in the older age categories into 18–29 y, 30–39 y, 40–49 y, and 50 y or older. 

#### 2.2.2. Depression and Anxiety Self-Reported Diagnosis

Diagnoses of depression and anxiety were self-reported through a question that asked about existing diagnoses of chronic diseases: “Has a doctor ever told you that you had or have any of the following health conditions?” A checklist of over 30 health conditions followed, which included anxiety and depression [25], among other physical conditions. For each disease the participant selected “yes” if they had been previously diagnosed; otherwise, it was left blank and categorized as “no”.

#### 2.2.3. Covariates

The covariates assessed for the study included sex, education, marital status, income, diagnoses of chronic diseases, and smoking status, which were all self-reported as part of the online survey. Sex response options were “male”, “female”, and “prefer not to answer”. Participants self-reported whether they were “married”, “living with a partner”, “divorced”, “single”, “separated”, or “widowed”, and the response options were further collapsed into “married/living with a partner” vs. “divorced/single/separated/widowed”. Participants also reported their highest level of education and current annual household income. Education categories were collapsed into “high school graduate or less”, “associate degree”, “some college”, “undergraduate degree”, “master’s degree”, and “doctoral degree”. Income categories were further collapsed into “≤20,000 USD”, “20,001–40,000 USD”, “40,001–75,000 USD”, and “>75,000 USD”. For chronic diseases, participants were asked to report if they had been diagnosed with any chronic disease listed, which included cardiovascular, cardiometabolic, respiratory, autoimmune, endocrine, autoimmune, rheumatic, cancer, kidney, and liver diseases. These were further collapsed into a binary variable (none vs. any disease). Lastly, current smoking was assessed with the self-reported question “Do you currently smoke?”, with the response options being “yes” or “no”. 

### 2.3. Statistical Methods

Descriptive statistics for the participant characteristics were expressed using frequencies and percentages for the total samples. Differences in participant characteristics by age group were tested using chi-square tests. Multivariate logistic regression models were used to estimate the odds ratios (OR) and corresponding 95% confidence intervals (95% CI). Models were adjusted for sex, education, income, marital status, chronic diseases, and smoking, all entered simultaneously into the models as categorical variables. STATA version 14 (StataCorp LLC, College Station, TX, USA) was used for all analyses. The significance level was set at *p* < 0.05. 

## 3. Results

Overall, 25% of the samples were between 18 and 29 y, 19% between 30 and 39 y, 22% between 40 and 49 y, and 34% ≥50 y (Table 1). Of the total sample, most participants were female (76%) and married/living with a partner (56%). Additionally, about 36% had an undergraduate degree, 25% had an annual household income between 20,001 USD and 40,000 USD, and a very small proportion (6%) reported smoking. In all, approximately one-fourth (23.6%) of participants reported having an anxiety diagnosis, whereas one-sixth (16%) reported a depression diagnosis. Differences in specific characteristics were observed by age group (Table 1). For the 18–29 y age group, a greater proportion had an associate or undergraduate degree, had an annual income of <20,000 USD, and were more likely to not be married or living with a partner. Additionally, the proportion of persons with chronic diseases was significantly higher as the age of the groups increased. Those currently smoking were more frequent in the 40–49 y and ≥50 y age groups.

In bivariate analyses, there were significant differences in the self-reported anxiety diagnosis across the age groups (Table 1). A higher proportion of persons in the youngest age group reported an anxiety disorder (18–29 y: 30%) compared to the older age groups (30–39 y: 23.7%, 40–49 y: 23.0%, and >50 y: 19.2%; *p* < 0.001). A self-reported depression diagnosis was only marginally significantly higher in the 18–29 y (18.1%) and ≥50 y (17.2%) groups than in the remaining age groups (30–39 y: 14.9% and 40–49 y: 14.9%). Similar results were observed in the adjusted logistic regression models (Table 2). Participants in the 18–29 y group (vs. ≥50 y) had 84% higher odds of reporting an anxiety diagnosis (95% CI = 1.34–2.55). In addition, those in the 30–29 y group had 50% (95% CI = 1.09–2.07) higher odds of reporting an anxiety diagnosis than those in the ≥50 y group, whereas those in the 40–49 y age group had 37% higher odds (95% CI = 1.01–1.87). No association between age and depression diagnosis was observed in all age groups.

## 4. Discussion

### 4.1. Summary of Findings

This study showed that both self-reported depression and anxiety diagnoses were common among adults in Puerto Rico during the COVID-19 pandemic and that younger adults (18–29 y, 30–39 y, and 40–49 y) experienced a disproportionate burden of anxiety compared to older adults (≥50 y).

The estimates of anxiety and depression symptoms during the pandemic (May 2020–August 2021) in the US were 20–30% and 28–37%, respectively [26]. Our estimates of anxiety and depression are closer to the lower range. However, it is important to note that our study specifically assessed self-reported physician-diagnosed depression and anxiety, whereas the US estimates are based on measures capturing anxiety and depressive symptoms (GAD-2 and PHQ-2). Further research using the same measures to assess anxiety and depression disorders is needed to make comparisons across different studies. The high estimates of an anxiety diagnosis observed in our sample may also reflect the past and ongoing experiences with chronic stressors (i.e., limited resources, high poverty, natural disasters and their delayed recovery, and a 15-year-long economic crisis) that may have worsened during the pandemic; thus, studies evaluating contributors to mental health disorders in this vulnerable population are needed. 

Our findings of a self-reported anxiety diagnosis being higher among younger adults during the pandemic are in agreement with previous studies conducted worldwide [4,5,6]. Research suggests that factors such as financial instability and loneliness greatly impacted younger adults and increased their anxiety rates compared to older adults [27]. In addition, a study in the mainland US documented that younger people reported greater pandemic-related stress, more life changes, higher social isolation, and lower quality of relationships than older people [6]. Critical for younger adults who may be at the crucial life stage of gaining financial independence, the pandemic created economic and job uncertainty [28], which may also negatively impact anxiety [29]. It is important to highlight that, given Puerto Rico’s unique sociocultural and political environment, including its relationship with the US, its recent exposure to several natural disasters (and their delayed recovery and subsequent humanitarian crises), and ongoing financial instability, its context differs from other countries, which makes it difficult to compare with other populations. 

The literature suggests plausible explanations as to why older adults may have presented lower levels of anxiety and depression than younger adults during the COVID-19 pandemic [30,31,32,33,34,35]. Research has documented that resilience in the elderly population may act as a buffer against negative experiences [30,31]. In addition, older adults may have fewer responsibilities and obligations, which can increase their sense of control [32]. Another potential reason is the documented higher level of social support networks observed among older adults, which may also help reduce the impact of stress and anxiety [31,33]. Lastly, older adults may have more experience dealing with challenging life events and may have developed effective coping strategies over time, which could have helped them better manage stressors during the pandemic [31]. On the contrary, social stigma around mental health outcomes in older vs. younger generations may act as a barrier to reporting depression and anxiety, resulting in underreporting among adults of older ages [34,35]. Thus, all these factors provide a plausible explanation for our findings of younger adults having a greater burden of anxiety. Nonetheless, the lack of association with depression may be due to the fact that, according to the data from the Behavioral Risk Factor Surveillance System in Puerto Rico, depression was higher among people 45 to 65 y during pre-pandemic years [36].

These findings have important public health implications. Firstly, public health campaigns, policies, and interventions need to focus on increasing accessibility to mental health services during emergencies, specifically for young adults. Secondly, research that evaluates the implementation of timely financial incentives and interventions that capitalize on important cultural values of the population in Puerto Rico (i.e., familism and social support) is needed. These may help build and improve social capital and decrease the burden of young adults’ experiences with anxiety as the population recovers from the pandemic [37]. Lastly, policies that ameliorate job insecurity across the public and private sectors during future public health emergencies should be evaluated to preserve the population’s mental health, particularly that of young adults.

### 4.2. Study Strengths and Limitations

The present findings need to be interpreted with caution and within the limitations of the study. First, the results are based on data that were collected through online recruitment, which may introduce selection bias, as reflected in the greater proportion of females and individuals of higher socioeconomic status in our sample [20,38,39,40]. Hence, our results may not be generalized to all residents of Puerto Rico. Future studies should recruit participants utilizing both in-person (using safety protocols) and online strategies to increase the rate of recruitment and participation of males of diverse socioeconomic status positions. Second, our study had a cross-sectional design; therefore, we could not establish causality or the onset of depression and anxiety throughout the pandemic. Future longitudinal studies are needed to examine this. Third, our study used a convenience sampling design, which may have resulted in a biased sample that is not representative of the adult population in Puerto Rico. Persons who are more willing or reachable to participate may have distinct characteristics from those who are not [41,42,43,44]. This leads to having an under- or overrepresentation of specific population subgroups, affecting the generalizability of the findings [43,44]. This was observed in our sample given the high proportion of females and people of higher education. Future studies should address sampling issues by utilizing multiple sampling methods to mitigate the limited generalizability of using convenience sampling methods [42,43,44]. Fourth, the study relied on self-reported diagnoses, which may have resulted in underreporting due to social desirability biases. However, studies have shown that self-reported diagnoses of mental health disorders and other chronic diseases are a good proxy for clinical diagnoses, as shown by a good percentage of agreement with clinical assessments [39,40,45]. Future studies should aim to validate self-reported diagnoses in Puerto Rico using clinical assessments [46] to ensure their adequacy in this group. Fifth, our study did not collect data on the prior history of mental health disorders. Without a prior history of diagnoses, we cannot document the pandemic’s true impact on mental health disorders. Thus, active surveillance during future public health emergencies is needed, particularly among young adults, to accurately estimate their impact on mental health disorders. Another study limitation is that information on medication use for the symptoms of mental health disorders was not collected [47]. This is an important confounder that future studies need to take into consideration. Lastly, our study did not include measures on other potential confounding variables, such as social support or exposure to COVID-19, all of which may be predictors of negative mental health outcomes [48,49,50,51,52]. Future studies should incorporate a broader set of confounding variables and better estimate the burden of mental health disorders. Despite these limitations, our study is the first to report that mental health disorders were common during the COVID-19 pandemic among adults in Puerto Rico and that the burden of anxiety differed by age group.

## 5. Conclusions

In conclusion, this study documented the burden of self-reported diagnoses of depression and anxiety disorders in a sample of adults residing in Puerto Rico during the pandemic and its variations across age groups. Our results evidenced differences in a self-reported anxiety diagnosis by age, with younger adults having a higher burden. Tailored public health campaigns and interventions by age groups are needed for future public health emergencies. 

## Figures and Tables

**Table 1:** Sample characteristics by age group among adults during the COVID-19 pandemic in Puerto Rico (n = 1945).

		Age Group				
	Total Sample (n = 1945)	18–29 y (n = 487)	30–39 y (n = 371)	40–49 y (n = 435)	≥50 y (n = 652)	*p*-Value
Sex ^a^						0.057
Female	1473 (76.4)	351 (72.5)	284 (77.2)	247 (80.1)	491 (76.2)	
Male	456 (23.6)	133 (27.5)	84 (22.8)	86 (19.9)	153 (23.8)	
Marital status						<0.001
Married/living with partner	1089 (56.0)	107 (22.0)	247 (66.6)	313 (72.0)	422 (64.7)	
Divorced/single/separated/widow	856 (44.0)	380 (78.0)	124 (33.4)	122 (28.0)	230 (35.3)	
Education level						<0.001
High school graduate or less	71 (3.7)	40 (8.2)	3 (0.8)	8 (1.8)	20 (3.1)	
Associate degree	169 (8.7)	102 (20.9)	14 (3.8)	16 (3.7)	37 (5.7)	
Some college	103 (5.3)	12 (2.5)	28 (7.6)	26 (6.0)	37 (5.7)	
Undergraduate degree	699 (35.9)	211 (43.3)	111 (29.9)	129 (29.7)	248 (38.0)	
Masters	549 (28.2)	96 (19.7)	121 (32.6)	152 (34.9)	180 (27.6)	
Doctoral degree	354 (18.2)	26 (5.3)	94 (25.3)	104 (23.9)	130 (19.9)	
Annual household income						<0.001
≤$20,000	327 (16.8)	157 (32.2)	41 (11.1)	40 (9.2)	89 (13.7)	
$20,001–$40,000	480 (24.7)	115 (23.6)	104 (28.0)	99 (22.8)	162 (24.9)	
$40,001–$75,000	473 (24.3)	86 (17.7)	96 (25.9)	108 (24.8)	183 (28.1)	
>$75,000	476 (24.5)	60 (12.3)	106 (28.6)	146 (33.6)	164 (25.2)	
Prefer not to answer	189 (9.7)	69 (14.2)	24 (6.5)	42 (9.7)	54 (8.3)	
Current smoking	114 (5.9)	15 (3.1)	18 (4.9)	32 (7.4)	49 (7.5)	0.006
Chronic diseases	1189 (61.1)	209 (42.9)	202 (54.5)	265 (60.9)	513 (78.7)	<0.001
Mental health disorders						
Anxiety	459 (23.6)	146 (30.0)	88 (23.7)	100 (23.0)	125 (19.2)	<0.001
Depression	310 (15.9)	88 (18.1)	45 (12.1)	65 (14.9)	112 (17.2)	0.081

^a^ Data missing for 16 participants. *p*-values are from chi-square tests.

**Table 2:** Association between age for anxiety and depression among adults during the COVID-19 pandemic in Puerto Rico.

	Age Group			
	18–29 y OR (95% CI)	30–39 y OR (95% CI)	40–49 y OR (95% CI)	≥50 y
Anxiety	1.84 (1.34–2.55)	1.50 (1.09–2.07)	1.37 (1.01–1.87)	1.00
Depression	1.05 (0.74–1.52)	0.78 (0.53–1.14)	0.95 (0.68–1.34)	1.00

Estimates are from adjusted logistic regression models (OR = odds ratio; CI = confidence interval). All models are adjusted for sex, marital status, education, income, diagnosis of chronic diseases, and smoking. No mental health disorder and age ≥50 y are used as the reference groups.

## Data Availability

The derived data supporting the findings of this study are available from the corresponding author upon request.
